# Fish Tank Granuloma Caused by *Mycobacterium marinum* in Two Aquarists: Two Case Reports

**DOI:** 10.1155/2013/161329

**Published:** 2013-12-12

**Authors:** Michal Slany, Petr Jezek, Monika Bodnarova

**Affiliations:** ^1^Veterinary Research Institute, Hudcova 70, 621 00 Brno, Czech Republic; ^2^Department of Clinical Microbiology and Parasitology, County Hospital Pribram, 261 95 Pribram, Czech Republic; ^3^Department of Dermatovenerology, General University Hospital and 1st Faculty of Medicine Charles University, 128 21 Prague, Czech Republic

## Abstract

*Mycobacterium marinum*, the cause of chronic systemic infections in fish, occasionally causes granulomatous skin and soft tissue lesions in humans. Cutaneous mycobacterial infection in two patients owing to unusual circumstances is presented in this report. The first patient was infected through improper hygienic behavior, while infection in the second patient was previously misdiagnosed as rheumatoid arthritis and treated with methylprednisolone for a period of three months, which resulted in a rare systemic spread of *M. marinum* into the bones of the hand, testis, and epididymis. Simultaneously, screening for possible sources of *M. marinum* infection in patients' aquaria revealed positive fish harboring VNTR profiles identical to those obtained for clinical isolates from patients.

## 1. Introduction

Nontuberculous mycobacteria (NTM) are ubiquitous in the environment and are responsible for several diseases in animals and humans known as mycobacterioses [1–3]. A study published in 2013 has shown that *M. fortuitum, M. gordonae, M. kansasii, *and *M. peregrinum *are frequently present in the aquatic environment of surface waters in the Moravian region of the Czech Republic. In contrast, the most frequent NTM present in aquaria with ornamental fish were *M. marinum* [4].

*Mycobacterium marinum* is the cause of chronic systemic infections in fish and an occasional cause of granulomatous skin lesions in humans. Infections in humans result in skin and soft tissue infections characterized by their predilection for the upper extremities, often following minor trauma to hands with a history of typical exposure to aquarium tanks [5, 6]. Diagnosis is usually made after biopsy and culture of the lesion, but microbiologists should be alerted to the possibility of *M. marinum* if the injury originated from an aquarium or the water environment [7].

Here we report two cases of *M. marinum* infection in humans (Table 1). Previously described species-specific qPCR targeting the *erp* and IS*2404* genes together with a conventional culture method was used for the detection of *M. marinum* in clinical specimens of two infected humans (Table 2). Simultaneously, epidemiology screening for possible sources in patients' aquaria via VNTR fingerprinting of *M. marinum* isolates was carried out.

## 2. Materials and Methods

### 2.1. Sample Collection

A total of 49 samples were taken from two humans (*n* = 9), ornamental fish (*n* = 20), and the aquarium environment (*n* = 20; biofilms, water, and plants). Animal and environmental samples were examined within the framework of an epidemiological study carried out in the aquaria of infected aquarists.

### 2.2. Cultivation and Isolate Characterization

All fish and environmental samples were subjected to decontamination with N-acetyl-L-cysteine followed by cultivation at 25°C, 30°C, and 37°C for 8 weeks [8]. In case of clinical samples decontamination step was excluded. Mycobacterial isolates were grown on solid Löwenstein-Jensen and Ogawa media (Trios, Prague, Czech Republic) and in liquid Sula medium (Trios). Clinical specimens were subjected to microscopic examination after Ziehl-Neelsen (ZN) staining for the detection of acid fast bacilli (AFB).

Mycobacterial isolates were identified by their phenotypic properties (temperature preference, growth rate, and pigment production in the dark and upon exposure to light), microscopic examination after ZN staining, and sequence analysis of the *16S rRNA* gene [9]. Subsequently, VNTR analysis according to a previously published method was used to fingerprint isolates identified as *M. marinum* [10].

### 2.3. DNA Isolation and Molecular Detection of *Mycobacterium marinum*

DNA isolation from human, fish, and environmental samples was based on a protocol described previously [11]. The isolated DNA was analyzed according to a previously described *erp*/IS*2404* qPCR assay, which enables species-specific detection of *M. marinum* [12].

## 3. Patient Clinical Presentation

### 3.1. Patient 1

A Twenty-five-year-old male reported cleaning his injured left knee with a brush usually used to clean the aquarium, which resulted in infiltration and subsequently visible crusts together with two subcutaneous resistances caused by *M. marinum*. The patient was initially treated with clarithromycin. Subcutaneous resistance did not lessen after nine weeks; therefore, treatment was continued with ethambutol for an additional nine weeks.

### 3.2. Patient 2

The second case involved cutaneous infection of a 60-year-old male with a professional interest in fish breeding. He reported injury to the tip of his index finger, which occurred during aquarium clearing. He was misdiagnosed with rheumatoid arthritis and treated with methylprednisolone for a period of 3 months, which resulted in a rare systemic spread of *M. marinum* from the primary site into the joint and bones of the index and ring finger, forearm, testis, and epididymis. The patient was initially treated with a combination of clarithromycin and ethambutol. Because of the insufficient clinical response in the area of the right hand an eight-month-long treatment based on results of sensitivities to a combination of amikacin, ciprofloxacin, ethambutol, cycloserine, and pyrazinamide was administered. Due to the damage to the testis an orchiectomy was also carried out. Because of long-lasting pain and damage the distal part of the ring finger was surgically removed.

## 4. Results and Discussion

Generally, three main histopathological patterns of *M. marinum* infection are distinguished: granulomatous nodular or diffuse inflammation with mixed granulomas; abscesses with mild granulomatous reactions; and subcutaneous (patients 1) or deep dermal granulomatous inflammation [13]. Tenosynovitis, septic arthritis, bursitis, or osteomyelitis was also reported in the literature to follow from deep subcutaneous infection [14, 15]. *M*. *marinum* infection may have the potential for systemic dissemination in humans as has been reported earlier and was shown in the second patient [6].

Conventional microbiological methods used for *M. marinum* diagnostics are slow and rely solely on phenotypic characteristics, which can be very similar to different causative agents. Clinicians in general have to consider other causes besides *M. marinum* including deep fungal infection; therefore, a diagnostic ladder is needed in this kind of patients. Delayed diagnosis is the main cause of the patients' serious side effect. Rapid and accurate molecular diagnosis methods are essential. Rapid detection of mycobacteria using conventional broad range PCR assay was previously described in the literature [16]. The advantage of this approach is the ability to detect multiple mycobacterial species. Generally, conventional PCR could show weakness such as proneness to contamination or lack of sensitivity when used for detection in clinical specimens where pathogen is present in limited amount [17]. Sensitive and specific method for the direct detection of *M. marinum* in clinical specimens suitable to overcome this limitation would be beneficial. So far, there have been very few reports on the detection of *M. marinum* directly from infected tissue without previous culturing [12, 18].

The molecular based approach used in this study represents a fast, sensitive, and specific method for the detection of *M. marinum*, in comparison to more time-consuming conventional methods. The protocol used here enabled us to complete the analysis of a sample, including controls, in approximately 6 hrs. The applied qPCR assay enabled the detection of *M. marinum* in clinical specimens collected from both the infected humans prior to successful cultivation (Table 2). Rare systemic spread of *M. marinum* in the second patient was proven by qPCR, microscopy, and cultivation. Furthermore, direct qPCR detection was successful in the case of three culture-negative samples.

Screening for the presence of *M. marinum* in the aquarium environment of both patients was carried out. The qPCR analysis of the patients' ornamental fish and aquarium environment revealed positivity for* M. marinum* in eight fishes (*n* = 3, patient 1; *n* = 5, patient 2). The number of viable cells in qPCR-positive samples was possibly lower due to the applied decontamination procedure because only one fish from each patient was later culture-positive for *M. marinum*. As reported earlier, microbiological stains give positive results in less than 25% of cases and do not correlate with the severity of *M. marinum* infection [14]. Although *M. marinum* is described to be present in the water environment, it was detected only in fish.

From the epidemiological aspect it has been proven that the *M. marinum* strain isolated from fish had the same VNTR profile as the clinical isolate originating from the fish owner and thus it can be concluded that the aquaria were the source of infection in the case of both aquarists (Table 3).

## 5. Conclusion

With regard to health, it is important that many NTM species classified as potentially pathogenic survive not only in the water environment, but also in infected fish. Contact zoonosis may occur particularly in risk groups such as aquaculture and fishery professionals, fish processors, ornamental fish hobbyists, and also consumers. The diagnosis of infections is usually hampered by the unfamiliarity of clinicians with disease agents derived from aquatic species. Fish tank exposure is the source of most cases of cutaneous *M. marinum *infections. It should be considered as a possible threat to humans reporting close contact with this environment and may be prevented by the use of waterproof gloves by persons with acute or chronic open skin lesions.

## Figures and Tables

**Table 1:** Cutaneous infection of* Mycobacterium marinum* in two patients.

Patient no.	Age/sex	Case background	Drug therapy		
			Regimen	Course	Treated
(1)	25/m	Hobby aquarist who cleaned injured knee with a brush usually used to clean the aquarium	Clarithromycin	Infiltrate with visible crusts together with two subcutaneous resistances on the left knee Extirpation of subcutaneous nodule because positivity for *M. marinum* in the sample of exploratorily excised cutaneous tissue was not detected	Yes
			Clarithromycin Ethambutol	Treatment changed due to the insufficient clinical response in the area of subcutaneous resistance	

(2)	60/m	Patient with a professional interest in fish breeding reported finger injury during aquaria cleaning	Methylprednisolone Methotrexate	Suspected rheumatoid arthritis due to swelling of the index finger joint Immunosuppression contributed to the hematogenous spread of infection	Yes
			Clarithromycin Ethambutol	Insufficient clinical response in the area of the right hand	
			Amikacin Ciprofloxacin Ethambutol Cycloserine Pyrazinamide	Severe right-hand oligoarthritis Osteomyelitis of the affected metacarpophalangeal joints Orchiectomy due to inflammation and necrosis in the epididymis with continuous spreading to the testis Surgical removal of the ring finger	

**Table 2:** The presence of *Mycobacterium marinum* in clinical specimens studied using the *erp* and IS*2404* qPCR assay.

Sample		*erp*/IS*2404* qPCR	ZN microscopy	Cultivation	Isolate identity
Matrice	Source				
Patient 1					
Subcutaneous nodule	Left knee	1.6 × 10^7^	AFB	+	*M. marinum *
Cutaneous tissue	Left knee	−	−	−	−

Patient 2					
Sperm	Testis	−	−	−	
Puncture	Metacarpal joint	2.8 × 10^5^	AFB	+	*M. marinum *
Pus	Ring finger	5.2 × 10^6^	AFB	+	*M. marinum *
Blood	Vein	6.2 × 10^3^	−	−	−
Subcutaneous nodule	Right forearm	4.1 × 10^5^	−	+	*M. marinum *
Tissue	Epididymis	2.8 × 10^7^	AFB	−	−
Tissue	Testis	1.2 × 10^5^	AFB	−	−

Quantification of *M.  marinum* is shown in genome equivalents per g of tissue or mL of liquid sample.

AFB: acid fast bacilli.

**Table 3:** VNTR analysis of *Mycobacterium marinum* isolates.

Isolate source	VNTR loci^a^								
	Locus 1	Locus 6	Locus 15	Locus 16	Locus 18	MIRU2	MIRU5	VNTR2067	VNTR3422
Patient 1									
Subcutaneous nodule	2	2	3	3	2	2	NA	3	4
Fish	2	2	3	3	2	2	NA	3	4

Patient 2									
Puncture	2	2	3	4	2	2	2	3	4
Pus	2	2	3	4	2	2	2	3	4
Subcutaneous nodule	2	2	3	4	2	2	2	3	4
Fish	2	2	3	4	2	2	2	3	4

^a^NA: no amplification. As Locus 9, Locus 14, and VNTR1132 failed in amplification from any isolates, they are not listed here.
